# Epigenetics of traumatic stress: The association of *NR3C1* methylation and posttraumatic stress disorder symptom changes in response to narrative exposure therapy

**DOI:** 10.1038/s41398-023-02316-6

**Published:** 2023-01-19

**Authors:** Sarah Wilker, Vanja Vukojevic, Anna Schneider, Anett Pfeiffer, Stefan Inerle, Markus Pauly, Thomas Elbert, Andreas Papassotiropoulos, Dominique de Quervain, Iris-Tatjana Kolassa

**Affiliations:** 1 Clinical Psychology and Psychotherapy, Bielefeld University, Universitätsstraße 25, 33615 Bielefeld, Germany; 2 vivo international e.V., P.O. box 5108, 78430 Konstanz, Germany; 3 Clinical and Biological Psychology, Ulm University, Albert-Einstein-Allee 47, 89081 Ulm, Germany; 4 Research Platform Molecular and Cognitive Neurosciences (MCN) , Department of Biomedicine, University of Basel, Birmannsgasse 8, 4055 Basel, Switzerland; 5 Psychiatric University Clinics, University of Basel, CH-4055 Basel, Switzerland; 6 Department of Statistics, TU Dortmund University, Joseph-von-Fraunhofer-Straße 2-4, 44227 Dortmund, Germany; 7 Research Center Trustworthy Data Science and Security, UA Ruhr, Joseph-von-Fraunhofer-Straße 25, 44227 Dortmund, Germany; 8 Clinical Psychology and Neuropsychology, University of Konstanz, Universitätsstr. 10, 78457 Konstanz, Germany

**Keywords:** Predictive markers, Genetics, Human behaviour

## Abstract

Epigenetic processes allow plasticity in gene regulation in response to significant environmental events. Accumulating evidence suggests that effective psychotherapy is accompanied by epigenetic changes, rendering DNA methylation a potential biomarker of therapy success. Due to the central role of glucocorticoid dynamics in stress regulation and the alteration of aversive memories, glucocorticoid receptors are likely involved in the molecular processes that are required to successfully treat Posttraumatic Stress Disorder (PTSD). This study aimed to investigate the relationship between methylation at the glucocorticoid receptor gene (*NR3C1*) and PTSD treatment success of evidence-based psychotherapy. A sample of *N* = 153 conflict survivors from Northern Uganda (98 females and 55 males) with PTSD were treated with Narrative Exposure Therapy (NET). Diagnostic interviews and saliva sampling took place at pretreatment and 4 and 10 months after treatment completion. We investigated potential associations between PTSD symptom development and methylation changes at 38 CpG sites spanning *NR3C1* over the three times of measurement using the repeated measures correlation. After accounting for multiple comparisons, DNA methylation at CpG site cg25535999 remained negatively associated with PTSD symptoms. These results were followed up by mixed models as well as structural equation modelling. These analyses revealed that treatment responders had a significant cg25535999 methylation increase after treatment with NET. Furthermore, lower methylation at cg25535999 pretreatment predicted a higher symptom improvement. Our results suggest different epigenetic profile dynamics at *NR3C1* cg25535999 in therapy responders compared to non-responders and underscore the central role of glucocorticoid signaling in trauma-focused therapy.

## Introduction

The repeated experience of extremely stressful events can result in the development of Posttraumatic Stress Disorder (PTSD). Intrusive, emotionally intense memories, which torment trauma survivors in the form of thoughts, pictures, nightmares, flashbacks, intense feelings or body sensations represent a hallmark of PTSD [1]. The modification of such aversive memory traces by means of exposure-based psychotherapy, such as Prolonged Exposure, Cognitive Therapy for PTSD, Eye Movement Desensitization and Reprocessing and Narrative Exposure Therapy (NET), has the highest evidence-base from controlled trials for the treatment of PTSD [2–4]. Of the aforementioned treatments, NET has particularly been developed to meet the needs of survivors of multiple and complex trauma, and can be effectively delivered by trained local health workers in low resource settings affected by humanitarian crises [5], such as Northern Uganda [6, 7].

Successful exposure-based therapy requires the reorganization of associative and contextual memories, thereby reducing the conditioned fear response to a stimulus that was previously associated with danger. During this process, an extinction memory is built up and competes with the original fear memory [1]. In exposure-based psychotherapy for PTSD, the trauma memory and the associated emotions, sensations, and physiological responses (i.e., the stress reaction) need to be activated by imaginary exposure to allow for their modification [8, 9]. The hypothalamus-pituitary-adrenal (HPA) axis is centrally implied in the stress reaction as well as in memory formation, retrieval and extinction. In more detail, glucocorticoids prepare the body for an adaptive response in life-threatening situations [10]. At the same time, binding of glucocorticoids to glucocorticoid receptors (GRs) mediate the termination of the stress reaction via a negative feedback loop [10]. Further, glucocorticoids facilitate the consolidation of new emotional memories and extinction learning, but inhibit memory retrieval [1, 11]. Due to the central role of glucocorticoids in the stress reaction as well as the development and extinction of aversive memories, they are likely to also be crucially involved in PTSD etiology and its treatment by means of exposure-based psychotherapy [12, 13].

While evidence regarding changes in basal cortisol levels and their relation to PTSD is mixed and seems to depend on many influencing factors including the timing of sample collection, the tissue studied and the time of trauma exposure [14], the majority of studies indicates an increased glucocorticoid receptor sensitivity in PTSD, particular in the central nervous system and the immune system [15–17]. In this line, genetic variations in the gene encoding the GR (*NR3C1*) were found to be robustly associated with PTSD risk in a recent meta-analysis [18].

In recent years, research increasingly focused on epigenetic modifications of *NR3C1* in response to life stress and PTSD psychopathology, which could result in altered GR expression. The majority of studies found evidence for an association between reduced cytosine methylation of the *NR3C1* promoter region and PTSD risk [19–22], that was in turn associated with increased expression of the GR [23]. However, it has to be noted that in a first longitudinal study, development of PTSD after deployment was not significantly associated with epigenetic changes at the *NR3C1* promoter after correction for multiple comparisons of the 52 sites investigated [24]. Further, to the best of our knowledge, so far only one pilot study investigated whether epigenetic alterations of *NR3C1* impact the outcome of exposure-based psychotherapy for PTSD and/or are reversible by effective treatment [25]. Therapy responders (*N* = 8) showed higher *NR3C1* promoter methylation at pretreatment compared to non-responders (*N* = 8). However, treatment response was not associated with significant methylation changes at the *NR3C1* site in this small sample. Due to the central role of glucocorticoid signaling in extinction learning and stress response regulation, the association of *NR3C1* methylation and response to exposure-based psychotherapy warrants further investigation.

In general, research regarding methylation changes in response to psychotherapy is scarce, but the first results are promising. Kumsta [26] summarized first evidence regarding epigenetic alterations in response to psychotherapy across different disorders. While the research methods, therapy approaches and genes under investigation differed between the six studies included in the review, a commonality of all studies was that responders and non-responders differed in the methylation changes in response to psychotherapy. This renders methylation changes a potential biomarker of therapy response [26]. The present study therefore aimed to investigate whether PTSD symptom improvement following exposure-based treatment by means of Narrative Exposure Therapy (NET) is accompanied by *NR3C1* methylation changes.

## Patients and methods

### Sample

The sample for this study was derived from a large treatment study with survivors of the war between the rebel group Lord’s Resistance Army (LRA) and the governmental forces in Northern Uganda cf [6]. We analyzed all study participants who were included into the treatment study (inclusion criteria: diagnosis of PTSD according to DSM-IV, no signs of severe current alcohol or drug abuse, no clinical signs of an acute psychosis, no psychotropic medication) with valid epigenetic data at the pretest and at least one follow-up assessment. In total, the resulting sample consisted of *N* = 153 individuals (98 females and 55 males), with a mean age of 32.45 (SD = 8.77). Of this sample, *N* = 2 individuals had missing epigenetic data for the first follow-up assessment, while *N* = 4 had missing epigenetic data for the second follow-up assessment. Following the recommendations for power calculations by Bakdash and Marusich [27], our sample was adequately powered to detect associations between PTSD symptoms and *NR3C1* methylation for an effect size of at least |ρ | = 0.2.

### Diagnostic interviews

Diagnostic interviews were conducted by intensely trained local interviewers. The interview training included general quantitative data collection methods as well as the concept of PTSD and relevant differential diagnoses. An event-list adapted for the local context was employed in order to assess the number of traumatic event types experienced and in order to identify the worst traumatic experiences [28]. We further employed the Posttraumatic Diagnostic Scale (PDS; [29]) as an interview in order to assess the diagnosis and severity of PTSD according to DSM-IV. Depressive symptoms were assessed by the respective section of the Hopkins Symptom Checklist (HSCL; [30]). All study instruments were translated into the local language (Luo), followed by blind back translations and group discussions. The interviews took place prior to the beginning of NET treatment (t_1_), and four (t_2_) and ten months (t_3_) after the end of treatment.

### NET treatment

In brief, NET is an exposure-based psychotherapy for PTSD that aims at chronologically reconstructing the autobiographical memory of trauma survivors and was particularly developed for survivors of multiple and complex trauma [9, 31]. After obtaining an overview of the client’s life story by means of the lifeline exercise, the following sessions entail exposure therapy to the most stressful traumatic experiences of the client. In these sessions, the therapist guides the client to connect the fragmented emotional, cognitive, sensory and interoceptive memories of the traumatic events with the corresponding context information. In the process of NET, a detailed chronological narration of the survivor’s life story is developed, which focusses on recounting the most arousing experiences across the entire life-span. For more details regarding the treatment, the reader is referred to Schauer et al. [9].

NET treatments were provided by local lay mental health workers under supervision of expert psychologists according to a field version of the treatment manual [9]. Treatment quality was assured by an in-depth training of all study therapists which included the theoretical concepts of NET as well as the practical implementation that was trained intensely in role-plays. Furthermore, treatment fidelity was monitored in weekly supervision meetings with expert psychologists, as well as by a detailed review of the treatment documentation. On average, participants received 12 sessions of NET.

The study procedures were approved by the Institutional Review Board of Gulu University, the Lacor Hospital Institutional Research Committee, the Ugandan National Council for Science and Technology, Uganda, the ethics committee of the German Psychological Society (Deutsche Gesellschaft fur Psychologie), and the ethics committee of the University of Konstanz. All participants provided written informed consent prior to participation.

### Epigenetic analyses

#### DNA isolation

Saliva DNA was collected using an Oragene DNA Kit (DNA Genotek, Ottawa, ONT) and initially extracted using the precipitation protocol recommended by the manufacturer. High-purity DNA was obtained by additional re-purification. For this purpose, 2 µg of DNA isolated via the Oragene procedure was incubated overnight at 50 °C with proteinase K (lysis buffer: 30 mM Tris-HCl pH 8.0, 10 mM EDTA, 1% SDS, 150 ng/l proteinase K), agitated by gentle orbital shaking. Next, the DNA was purified using a Genomic DNA Clean & Concentrator Kit (Zymo Research, Irvine, CA). The DNA quality and concentration were assessed using spectrophotometry (Nanodrop 2000; ThermoScientific, Waltham, MA) and fluorometry (Qubit dsDNA BR Assay Kit, Invitrogen, Carlsbad, CA), respectively.

### Infinium EPIC 850 K BeadChip methylation analyses

DNA isolated from the saliva samples was investigated with the Infinium Human Methylation EPIC 850 K array (Illumina, Inc., San Diego, CA). All subjects were processed in a single batch, with a single bisulfite conversion, and with balanced randomized plate assignment.

During pre-processing, data were extracted and analysed from the generated idat files using the R package RnBeads version 0.99.9 [32]. CpG annotation was based on the manufacturer’s annotation file (Infinium MethylationEPIC v1.0 B5 Manifest File). During pre-processing, the background was subtracted using the “noob” method in the methylumi package version 2.42.0; [33] and the signal was further normalized using the SWAN algorithm [34]. The following probe categories were excluded from the final data sets, based on the annotation provided within the RnBeads package: non-CpG context probes due to underrepresentation, [35], functional differences when compared to the CpG context as well as very low abundance of non-CpG methylation in somatic tissues [36], probes with a SNP mapping directly to the target CpG site; gonosomal probes; non-specific probes. Using the Greedycut algorithm, we iteratively removed the probes and data sets of the highest impurity (rows and columns in the detection *p*-value table that contain the largest fraction of unreliable measurements; *p* < 0.05; for each sample [32].

The B-values were further post-processed step-by-step in order to correct for further influential and putative confounding factors: 1) using logit-transformation, M-value [37] done with the R-package car [38] 2) z-transformation per plate (correcting for plate and batch effects); 3) regressing out the first 11 axes of a principal component analysis (PCA, done with the R-package pcaMethods [39]). The PCA was based on CpGs with no missing values (> 95% of the included CpGs). This approach additionally corrected for technical biases as well as for part of the variability induced by heterogenous cell composition. The given number of axes to regress out was based on a larger cross-sectional sample from the same population that included 47 technical replicate pairs [40] using a previously described quantitative trait loci driven approach [41] 4) regressing out the effects of sex and age. The accepted missing rate per CpG was set to < 1%. Only samples and CpGs surviving all filtering steps were used for the downstream analyses.

Finally, a re-measurement quality assessment was made based on the 47 technical replicate pairs as described above. For the downstream analysis we applied a relatively lower re-test threshold of r = 0.2, given that the *NR3C1* includes many sites with methylation values from the extreme regions of the bimodal distribution [41]. This resulted in the final dataset of N = 38 CpGs annotated to the *NR3C1* gene that were included in the downstream analysis.

### Statistics

All statistical analyses were conducted using the statistical environment R version 4.1.0 [42]. Model assumptions were checked by visual inspection of scatter plots and residual plots. We initially evaluated the PTSD symptom change in the whole therapy sample using mixed models with the PDS score as the outcome variable and time as a fixed effect. In order to further investigate the PTSD symptom changes between the three points of measurement (t_1_: before treatment; t_2_: 4 months after treatment and t_3_: 10 months after treatment) we calculated planned contrasts with corrections for multiple comparisons using the R package multcomp version 1.4-17 [43]. The analyses were repeated with depressive symptoms (HSCL depression score) as the outcome variable.

### Identification of CpG sites associated with treatment response

In order to identify associations between the change of PTSD symptoms in response to NET on the one hand, and potential corresponding epigenetic alterations on the other hand, we calculated the Repeated Measures Correlation (rmcorr; [27]; R Package rmcorr version 0.4.3). rmcorr determines the common intra-individual relationship between pairs of repeated measurement. Thereby, the best linear relationship between the two repeated measurements is calculated assuming varying intercepts but the same regression slope for each study participant. In the case of our data, this implies that rmcorr computes the association between the paired repeated measures, i.e., PTSD symptoms and methylations, and reveals if there are similar correlations for each individuum. Like the Pearson correlation coefficient, the absolute value of rmcorr can vary between 0 (no relationship) and 1 (perfect relationship). rmcorr was calculated for each investigated CpG site, and the resulting *p*-values were corrected for multiple comparisons using the FDR correction. CpGs with an FDR of < 0.05 were further investigated in subsequent analyses.

### Linear mixed models

The identified CpG site was further investigated using linear mixed models with methylation level as the outcome variable, time as a fixed factor and study participants as a random effect using the R package nlme version 3.1-152; [44]. Following a recent review regarding epigenetic alterations in response to psychotherapy [26] who noted that a strikingly similarity in epigenetic studies on therapy outcome were different epigenetic alterations in responders and non-responders, we analyzed the data for the whole sample, as well as for responders and non-responders separately.

Responders were defined as individuals with a symptom decline of > 5.37 from pretest (t_1_) to the last follow-up (t_3_). This cutoff was identified as a clinically significant change according to the reliable change index in the same population in a previous study by Schneider et al. [6]. Accordingly, only individuals with complete data points at the last follow-up (*N* = 149) were classified as responders or non-responders. The sample of responders included *N* = 116 individuals, whereas *N* = 33 individuals were classified as non-responders.

### Structural equation modelling

Finally, in order to get first hints regarding any potential directional relationship between PTSD symptom change and methylation change over time, we conducted cross-lagged analyses using structural equation modelling (SEM) implied in the package lavaan version 0.6-8 [45]. In cross-lagged designs [46], a variable measured at a later time can be predicted by a variable assessed at an earlier time. The comparison of these lagged associations can provide first hints on potential predictive relationships [47, 48].

The SEM included PTSD symptom severity and *NR3C1* methylation at each time point (t_1_, t_2_, and t_3_). Auto-regressive paths were added from each previous assessment of a given variable (e.g. PTSD symptoms assessed at t_1_) to the consecutive time point (e.g., PTSD symptoms assessed at t_2_). Further, we allowed correlations between PTSD symptoms and *NR3C1* methylation levels at the same time point. Finally, the cross-lags were included, which modeled the longitudinal relationship between PTSD symptoms and *NR3C1* methylation. The parameter estimates were calculated by means of maximum likelihood estimation. Since the assumption of multivariate normality was not met, robust standard errors were reported and model test statistics were Satorra-Bentler scaled as recommended by Rosseel [45].

## Results

Before treatment, the mean PDS score was 16.67 (SD = 4.66). PTSD symptoms declined after the treatment with NET (mean PDS score four months after treatment = 7.87 [SD = 5.51]; mean PDS score ten months after treatment = 6.83 [SD = 5.31]). In linear mixed models, we found a significant effect of time (*F* = 175.79, *p* < 0.0001). Treatment response was not influenced by time and sex (i.e., we did not find evidence for time × age or time × sex interaction effects). Thus, for reasons of parsimony, these covariates were not included in the downstream statistical analyses. Planned contrasts revealed a significant symptom decline between t_1_ and t_2_, t_2_ and t_3_, as well as between t_1_ and t_3_. According to the conventions of Cohen, the symptom decline from pretest to the last follow-up can be considered as large (*d* = 1.97). A significant symptom decline was also observed for depressive symptoms (*F* = 29.20, *p* < 0.0001) with a medium effect size (*d* = 0.70).

In order to investigate whether this symptom improvement was associated with *NR3C1* methylation changes, we calculated the repeated measurement correlation between the variables PDS Score and methylation at each CpG site. After applying FDR correction for multiple comparisons, the methylation at CpG site cg25535999 was significantly negatively correlated with the PDS Score across the three repeated measurements (rmcorr = −0.195, *p* = 0.0007, *FDR* = 0.025, cf. Supplemental Table 1 and Supplemental Fig. 1). Similarly, a negative association between cg25535999 and depressive symptoms was found across the three repeated measurements (rmcorr = −0.14, *p* = 0.014, *FDR* = 0.51). However, this association did not survive correction for multiple comparison and was therefore not followed up by subsequent analyses.

**Fig. 1:**
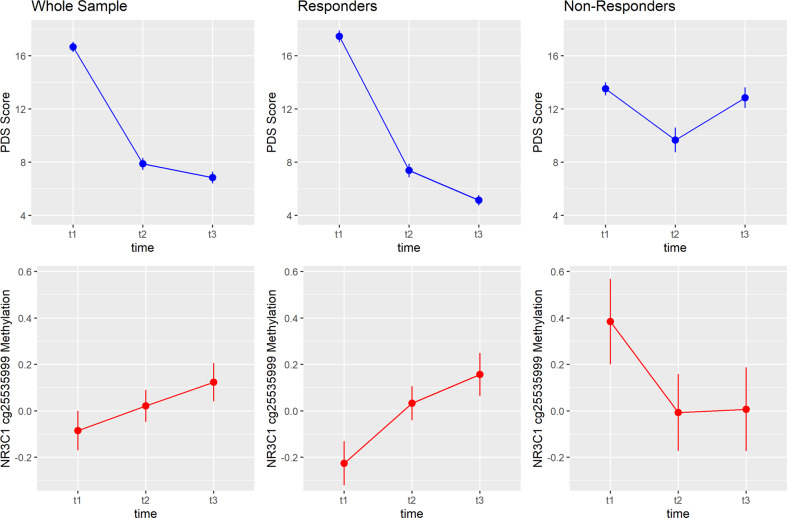
Mean values and standard errors of measurement for PTSD symptoms (upper panel) and *NR3C1* cg25535999 methylation (lower panel) over the three points of measurement. The plot separately displays the results for the whole sample (*N* = 153), responders (*N* = 116), and non-responders (*N* = 33). PDS, Posttraumatic Diagnostic Scale; t_1_, pretreatment; t_2_, 4 months posttreatment, t_3_, 10 months posttreatment.

We next calculated linear mixed models with cg25535999 methylation as the outcome and time as a fixed effect to further elaborate the relationship between symptom improvement and methylation changes. In the sample of therapy responders, cg25535999 methylation increased after NET (Fig. 1, F = 5.03, *p* = 0.007). Planned contrasts indicated a significant increase from t_1_ to t_2_ (*z* = 2.16, *p* = .04) as well as from t_1_ to t_3_ (*z* = 3.08, *p* = 0.003). The methylation increase from t_1_ to t_3_ corresponds to a small effect (*d* = 0.20).

In non-responders, a non-significant decrease of methylation was observed. Accordingly, the methylation increase in the whole sample was less pronounced than in the sample of responders and did not reach statistical significance. Thus, as illustrated in Fig. 1, the negative relationship between symptom development and methylation identified by the repeated measurement correlation is driven by the sample of therapy responders: the strong reduction of PTSD symptoms over time was accompanied by an increase of *NR3C1* cg25535999 methylation.

We did not find any differences between responders and non-responders regarding the number of traumatic events reported before therapy (*t* = 1.4284, *p* = 0.16), regarding depressive symptom severity before therapy (*t* = −0.94, *p* = 0.3534), or in the occurrence of new events after treatment (for t_2_: *Χ*² = 0.35, *p* = 0.56; for t_3_: *Χ*² = 1.51, *p* = 0.22).

**Fig. 2:**
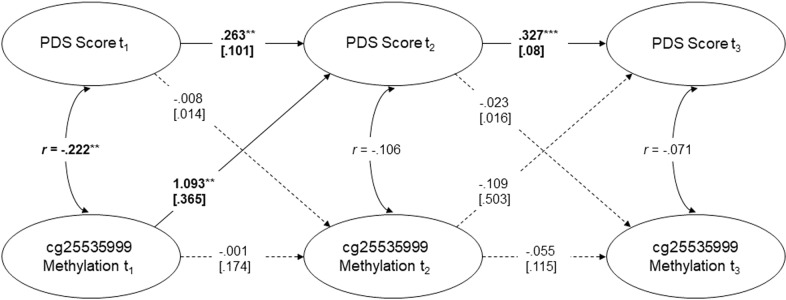
Displayed is the constructed Structural Equation Model (SEM) together with the regression or correlation coefficients. Significant associations are marked in bold. *p < 0.05, **p < 0.01, ***p < 0.001.

Finally, we employed a cross-lagged analysis in order to examine whether a model assuming predictive relationships between PTSD symptoms and methylation shows a good fit to the data. Furthermore, this model may contribute to answer the questions whether *NR3C1* cg25535999 methylation is predictive for PTSD symptoms and vice versa. The constructed SEM, visible in Fig. 2, had an excellent fit to the data with a root mean square error of approximation (RMSEA) of < 0.001 (90% - CI = [0, 0.105]), a comparative fit index (CFI) of approximately 1, and a non-significant deviation from the empirical covariance structure (*χ*^2^(4) = 2.915, *p* = 0.572) (cf. cut-offs proposed by Hu & Bentler who consider CFI > 0.95 and RMSEA < 0.06 as relatively good). We identified significant auto-regressive relationships for the PTSD symptoms assessed over time, but not for *NR3C1* cg25535999 methylation. Regarding the cross-lagged relationships, the only significant association was found between methylation status before therapy (t_1_) and PTSD symptoms four months after the end of treatment (t_2_) (*β* = 1.093, *SE(β)* = 0.365, *z* = 2.991, *p* = 0.003). Indeed, responders and non-responders differed significantly regarding cg25535999 methylation before therapy (*t* = 2.95, *p* = 0.005, cf. Fig. 1). No predictive influences were identified between t_2_ and t_3_.

### *NR3C1* cg255535999 methylation and GR expression

cg25535999 is located in the intron 7 of the gene body of *NR3C1* (see Supplementary Figure 2). In order to investigate the potential impact of cg25535999 methylation on *NR3C1* expression, we investigated publicly available data generated by The Cancer Genome Atlas (TGCA) project (https://www.cancer.gov/tcga), by using the SMART web-interface [49]. These analyses revealed that cg25535999 methylation is significantly correlated with *NR3C1* gene expression across 17 TCGA datasets. Further, the majority of the identified correlations was positive (for the distribution of Pearsons’ r across 17 TCGA datasets: μ = 0.41, *x*˜ = 0.43, σ = 0.22, see Supplementary Fig. 3). This finding agrees with the position of the given CpG site in the *NR3C1* gene body, and further corroborates the possible functional relevance of this epigenetic modification for *NR3C1* gene expression, and thus the regulation of glucocorticoid signaling in PTSD.

## Discussion

In line with a recent meta-analysis [5], we found evidence for a strong PTSD symptom reduction after treatment with NET. According to a cut-off of clinically significant change, the majority (78%) of our sample were classified as therapy responders. This sub-group showed a distinct epigenetic pattern: On the one hand, responders showed a significant *NR3C1* cg25535999 methylation increase after treatment with NET, which was not observed in non-responders. On the other hand, this group also presented with significantly lower methylation levels pre-treatment. This is in line with the evidence summarized by Kumsta [26], suggesting that therapy responders and non-responders show distinct epigenetic profiles that can be measured in the periphery. Our SEM indicated that pre-treatment DNA methylation of the cg25535999 site in the gene body of *NR3C1* was predictive of PTSD symptoms after NET treatment, with higher values in initial methylation being associated with higher PTSD scores after NET. One earlier pilot study also identified *NR3C1* methylation as a predictor of psychotherapy response [25], yet this studies focused on promoter methylation.

What might be the biomolecular mechanisms underlying our observations? First and foremost, it is important to note that the methylation differences observed in this study were measured in saliva samples. While we can assume that the consolidation of extinction memory is accompanied by epigenetic changes linked to synaptic plasticity in the brain [50], central changes are not necessarily reflected by similar changes occurring in the periphery [26]. Yet, one plausible mechanism how interventions are likely to impact methylation in peripheral cells are top-down effects on brain-body communication systems, i.e., the stress axes, which will stimulate physiological changes in the periphery [26, 50]. Accordingly, it is plausible to assume that the glucocorticoid receptor gene *NR3C1*, which holds a central function in the sensitivity of the HPA axis, could be subject to epigenetic changes in response to psychotherapy that can be measured in the periphery. Nevertheless, caution is generally required when interpreting epigenetic data in the periphery and premature conclusions about underlying processes in the brain should be avoided. Of course, this also has to be considered when interpreting the finding of a decreased *NR3C1* cg25535999 methylation status of treatment responders at pretreatment. Bearing that in mind, we found evidence that cg25535999 methylation correlated positively with *NR3C1* expression across different TGCA datasets. Accordingly, therapy responders, who had lower cg25535999 methylation before therapy, might also present with a lower expression of the GR before therapy, which could in turn be associated with higher levels of circulating cortisol in contrast to non-responders cf [23, 51]. These higher levels of cortisol might facilitate extinction learning processes during treatment [1, 52]. Therapy success was further accompanied by an increase in cg25535999 methylation, potentially associated with a higher expression of GRs and reduced cortisol levels. Follow-up studies should thus also include a direct measurement of *NR3C1* expression and cortisol related to the treatment timeline, in order to further substantiate the current findings.

Nevertheless, the results of this study highlight the central role of glucocorticoids in exposure-based treatments [52]. Further, they correspond with accumulating evidence indicating that the effectiveness of exposure-based therapy for varying anxiety disorders, including spider phobia [53, 54], social phobia [55] and fear of heights [56] is enhanced by glucocorticoid administration. Similar effects were observed in a pilot trial on exposure therapy for PTSD, however it has to be noted that the effect was likely mediated via a higher treatment retention rate in the glucocorticoid group as opposed to the placebo group [57].

### Limitations and future directions

The findings of this study have to be interpreted in light of its limitations. Similar to previous studies investigating epigenetic changes in response to psychotherapy, the study was of observational nature and did not investigate an additional or non-treatment patient control group. Accordingly, PTSD symptom changes as well as methylation changes cannot be causally attributed to the treatment.

Future randomized controlled trials are needed to substantiate findings regarding the relationship between treatment and methylation changes which can be adequately powered using the effect sizes reported in this manuscript. In addition, a higher number and density of assessments post therapy would be helpful to identify the chronological order of symptom and methylation changes.

Further, the epigenetic analyses were conducted on saliva samples. Therefore, the assessed epigenetic alterations may vary with a changed life-style, e.g., more sleep, altered physical activity etc. In particular conclusions regarding brain processes as well as overall HPA-functioning remain speculative. In this line, data on *NR3C1* expression and cortisol levels would have been helpful to interpret our findings. However, these biological samples are difficult to collect, process and store in a field study.

Finally, it has to be noted that glucocorticoid signaling is regulated by complex pathways. For instance, FKBP5, a co-chaperone of the GR, influences the negative feedback mechanisms of the HPA axis [58], and *FKBP5* methylation has been shown to be associated with PTSD symptom development [59, 60]. Future studies with even larger sample sizes might be adequately powered to investigate more complex epigenetic pathways including the interplay of several candidate genes.

## Conclusions

In sum, this study identified a distinct epigenetic profile of therapy responders in contrast to non-responders. Therapy response was predicted by lower *NR3C1* cg25535999 methylation at baseline, and correlated with a methylation increase. Our findings highlight the central role of glucocorticoids in relation to the effectiveness of trauma-focused therapy.

## Supplementary information

Supplemental Information

## Data Availability

Due to the highly sensitive nature of this research, participants of this study did not agree for their data to be made available in a data repository. However, participants gave their informed consent that excerpts of the data that guarantee anonymity can be shared with other researchers upon reasonable request. Please address requests to sarah.wilker@uni-bielefeld.de.

## Code availability

The R code can be requested from the corresponding author.
