# Supplementary material for: Epigenetics of traumatic stress: The association of NR3C1 methylation and posttraumatic stress disorder symptom changes in response to narrative exposure therapy
Source: Transl Psychiatry. 2023 Jan 19;13:14. doi: 10.1038/s41398-023-02316-6 (PMC9852425; doi:10.1038/s41398-023-02316-6)

**Supplemental Information**

Epigenetics of traumatic stress: *NR3C1* methylation is associated with Posttraumatic Stress Disorder symptom changes in response to Narrative Exposure Therapy

**Supplemental Table 1:** Results of the repeated measurement correlation between *NR3C1* methylation and PTSD symptoms over the three points of measurement

| CpG site | DF | rmcorr | p | FDR |
|---|---|---|---|---|
| cg25535999 | 299 | -0.195 | 0.00066 | 0.025 |
| cg19135245 | 299 | 0.13 | 0.02391 | 0.454 |
| cg19820298 | 297 | 0.11 | 0.05743 | 0.715 |
| cg16219186 | 299 | -0.095 | 0.09955 | 0.715 |
| cg24026230 | 299 | -0.091 | 0.11643 | 0.715 |
| cg03906910 | 299 | -0.088 | 0.12709 | 0.715 |
| cg19645279 | 299 | -0.087 | 0.13164 | 0.715 |
| cg20753294 | 299 | -0.072 | 0.21223 | 0.945 |
| cg18998365 | 299 | -0.07 | 0.22385 | 0.945 |
| cg18019515 | 299 | 0.066 | 0.25266 | 0.946 |
| cg13764763 | 299 | 0.063 | 0.27381 | 0.946 |
| cg15910486 | 299 | 0.056 | 0.33508 | 0.995 |
| cg26464411 | 299 | 0.047 | 0.41702 | 0.995 |
| cg00629244 | 299 | 0.047 | 0.41777 | 0.995 |
| cg19176661 | 299 | -0.042 | 0.47231 | 0.995 |
| cg12466613 | 299 | -0.04 | 0.48884 | 0.995 |
| cg05483455 | 299 | 0.037 | 0.52043 | 0.995 |
| cg15115787 | 299 | 0.037 | 0.52292 | 0.995 |
| cg20728768 | 299 | -0.034 | 0.55961 | 0.995 |
| cg08818984 | 299 | -0.031 | 0.5884 | 0.995 |
| cg03746860 | 299 | -0.031 | 0.58941 | 0.995 |
| cg07589972 | 299 | 0.027 | 0.63937 | 0.995 |
| cg17342132 | 299 | -0.026 | 0.65512 | 0.995 |
| cg23776787 | 299 | 0.022 | 0.7043 | 0.995 |
| cg08845721 | 299 | 0.021 | 0.71542 | 0.995 |
| cg12969488 | 299 | -0.015 | 0.79622 | 0.995 |
| cg24052866 | 299 | -0.014 | 0.80583 | 0.995 |
| cg12888360 | 299 | -0.014 | 0.80962 | 0.995 |
| cg18718518 | 299 | 0.011 | 0.84796 | 0.995 |
| cg00294552 | 298 | 0.01 | 0.8689 | 0.995 |
| cg07733851 | 299 | 0.008 | 0.8956 | 0.995 |
| cg17860381 | 299 | -0.007 | 0.90933 | 0.995 |
| cg08423118 | 299 | -0.005 | 0.93106 | 0.995 |
| cg23430507 | 299 | 0.003 | 0.96527 | 0.995 |
| cg22233604 | 299 | 0.001 | 0.98155 | 0.995 |
| cg08695103 | 299 | 0.001 | 0.98219 | 0.995 |
| cg03857453 | 299 | -0.001 | 0.99108 | 0.995 |
| cg01967637 | 299 | 0 | 0.99473 | 0.995 |

**Notes:** DF, Degrees of Freedom; rmcorr, Repeated Measurement Correlation; p, p-value; FDR, FDR-corrected p-value

**Supplemental Figure 1:** Scatterplot of Repeated Measurement Correlation (rmcorr) between PTSD symptom severity (PDS score) and methylation at *NR3C1* cg25535999 for each individual included in the treatment study. The three points of measurement for each individual are visualized in the same color, along with individual regression lines (assuming a random intercept and a fixed slope). PDS, Posttraumatic Diagnostic Scale.

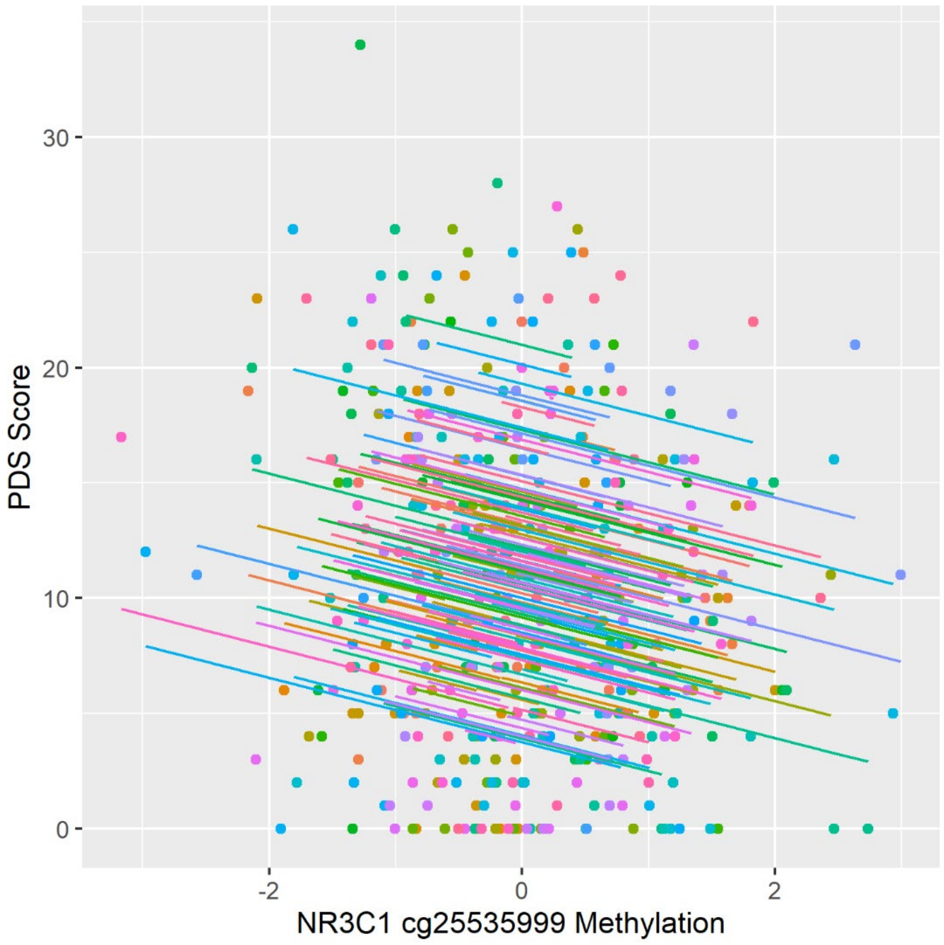

**Supplemental Figure 2:** The figure shows detailed chromosomal distribution of the *NR3C1* gene region in the SMART genome browser. The upper panel depicts the known Ensembl transcripts from the *NR3C1* gene locus, with transcript, exon, CDS and UTR regions marked red, blue, orange and green color, respectively. The yellow arrow marks the direction of transcription. The lower panel depicts the *NR3C1* gene locus with the locations of the Infinium CpG probes. DNA methylation at the encircled CpG site cg25535999 was significantly associated with PTSD symptoms over time, after accounting for multiple comparisons. CpG sites at density regions Island, N-shore, S-shore, N-shelf, S-shelf and NA are marked in red, orange, violet, blue, green and grey respectively.

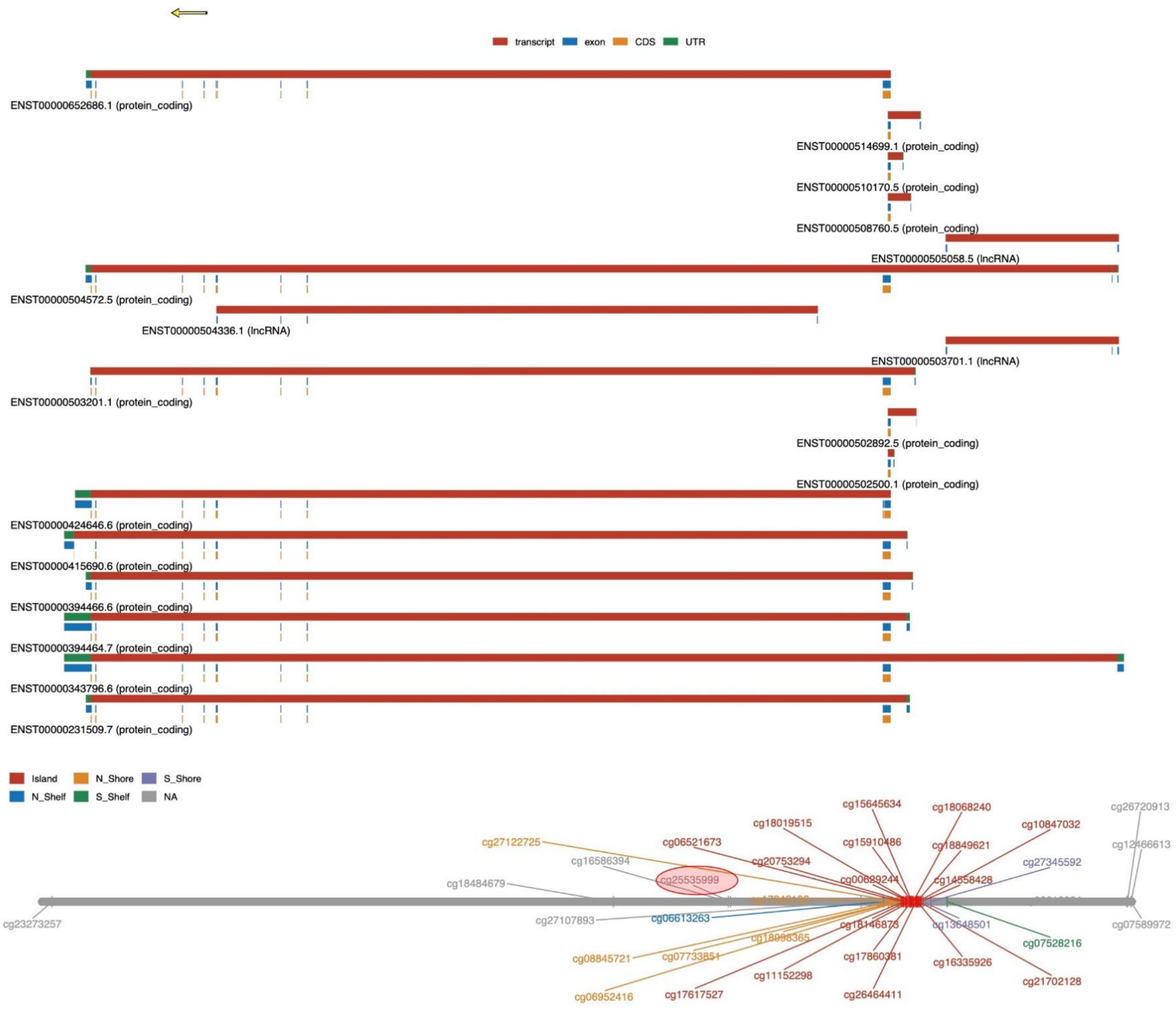

transcript
exon
CDS
UTR
ENST00000652686.1 (protein_coding)
ENST00000514699.1 (protein_coding)
ENST00000510170.5 (protein_coding)
ENST00000508760.5 (protein_coding)
ENST00000505058.5 (lncRNA)
ENST00000504572.5 (protein_coding)
ENST00000504336.1 (lncRNA)
ENST00000503701.1 (lncRNA)
ENST00000503201.1 (protein_coding)
ENST00000502892.5 (protein_coding)
ENST00000502500.1 (protein_coding)
ENST00000424646.6 (protein_coding)
ENST00000415690.6 (protein_coding)
ENST00000394466.6 (protein_coding)
ENST00000394464.7 (protein_coding)
ENST00000343796.6 (protein_coding)
ENST00000231509.7 (protein_coding)
Island
N_Shore
S_Shore
N_Shelf
S_Shelf
NA
cg15645634
cg18068240
cg26720913
cg18019515
cg10847032
cg27122725
cg06521673
cg15910486
cg18849621
cg12466613
cg16586394
cg20753294
cg27345592
cg25535999
cg00629244
cg14558428
cg18484679
cg23273257
cg27107893
cg06613263
cg18146873
cg13648501
cg07589972
cg18098365
cg07528216
cg08845721
cg07733851
cg17860381
cg16335926
cg11152298
cg21702128
cg06952416
cg17617527
cg26464411

**Supplemental Figure 3:** Correlation between *NR3C1* cg25535999 methylation and *NR3C1* expression across different TGCA datsets.

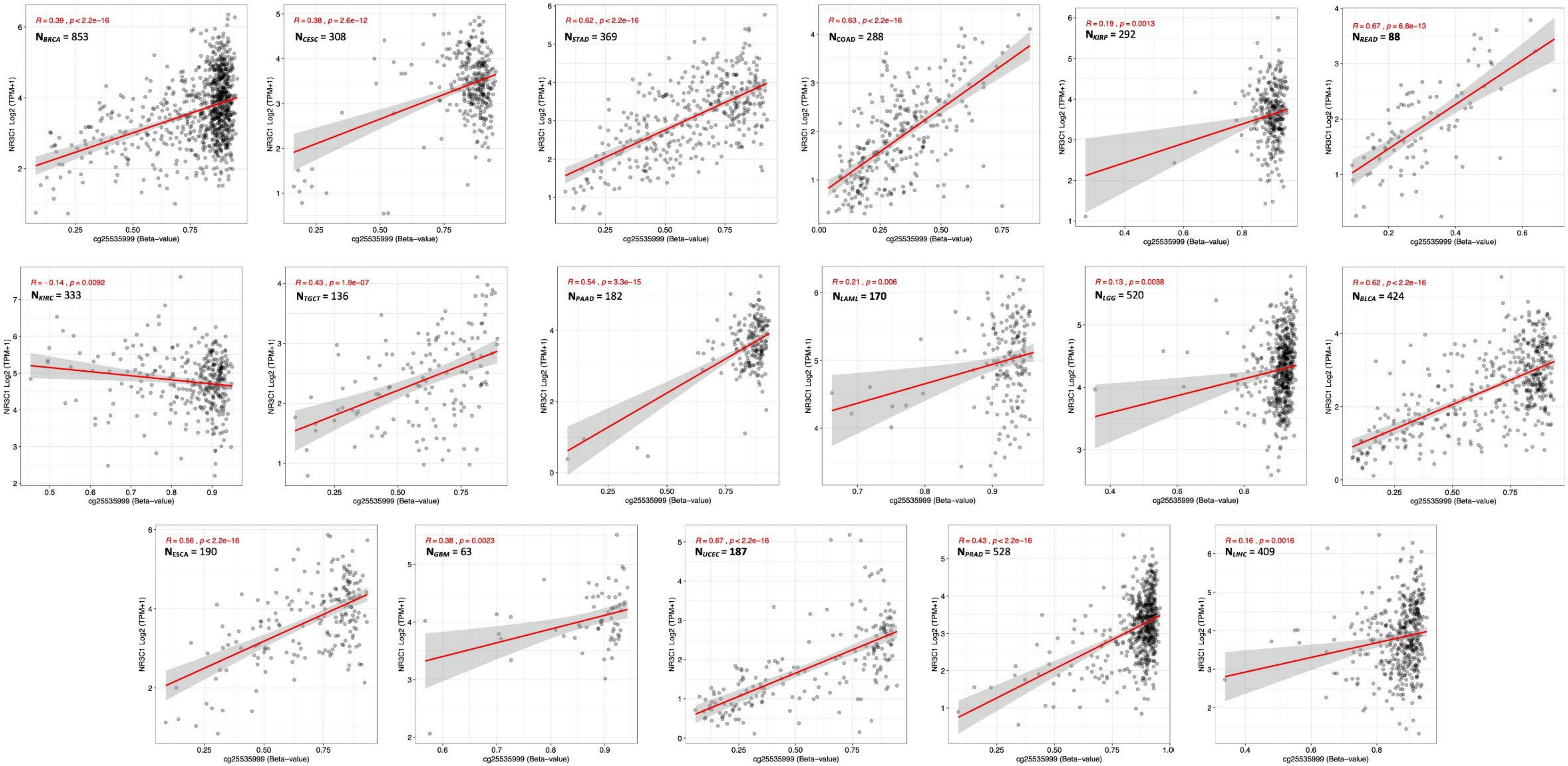

Supplement: Supplementary file 1 — Supplemental Information [file 41398_2023_2316_MOESM1_ESM.pdf]
